# Maternal exposure to benzophenone derivatives and their impacts on offspring's birth outcomes in a Middle Eastern population

**DOI:** 10.1038/s41598-023-35380-5

**Published:** 2023-06-10

**Authors:** Hakimeh Teiri, Mohammad Reza Samaei, Mansooreh Dehghani, Abooalfazl Azhdarpoor, Farzaneh Mohammadi, Roya Kelishadi, Yaghoub Hajizadeh

**Affiliations:** 1 Student Research Committee, Department of Environmental Health Engineering, School of Health, Shiraz University of Medical Sciences, Shiraz, Iran; 2 Department of Environmental Health Engineering, School of Health, Shiraz University of Medical Sciences, Shiraz, Iran; 3 Department of Environmental Health Engineering, Faculty of Health, Isfahan University of Medical Sciences, Isfahan, Iran; 4 Environment Research Center, Research Institute for Primordial Prevention of Non-Communicable Diseases, Isfahan University of Medical Sciences, Isfahan, Iran

**Keywords:** Developmental biology, Environmental sciences, Biomarkers, Risk factors

## Abstract

Widespread use of benzophenones (BPs), a group of environmental phenolic compounds, is suspected of interfering with human health. The association of prenatal exposure to benzophenone derivatives with birth outcomes including birth weight and length, head, arm and thoracic circumference, abnormalities, corpulence index and anterior fontanelle diameter (AFD) was investigated. Mother-infant pairs of 166 within PERSIAN cohort population in Isfahan, Iran, in the 1st and 3rd trimesters of pregnancy were assessed. Four common benzophenone metabolites including 2,4-dihydroxy benzophenone (BP-1), 2-hydroxy-4-methoxy benzophenone (BP-3), 4-hydroxy benzophenone (4-OH-BP) and 2,2′-dihydroxy-4-methoxy benzophenone (BP-8) were measured in maternal urine samples. The median concentration of 4-OH-BP, BP-3, BP-1 and BP-8 were 3.15, 16.98, 9.95 and 1.04 µg/g Cr, respectively. In the 1st trimester, 4-OH-BP showed a significant correlation with AFD in total infants, decreasing 0.034 cm AFD per a log unit increase of 4-OH-BP. Within the male neonates, 4-OH-BP in the 1st and BP-8 in the 3rd trimester were significantly associated with head circumference and AFD increase, respectively. Among female neonates in the 3rd trimester, increasing 4-OH-BP and BP-3 concentration was correlated with a decrease in birth weight and AFD, respectively. This study demonstrated that all the target BP derivatives can influence normal fetal growth at any age of the pregnancy, nevertheless, to support these findings further studies are needed in a large and different group population.

## Introduction

The advancement of science and technology parallel to the improvement of human welfare has imposed ubiquitous and emerging pollutants on the environment and consumable stuff which raised health concerns of exposure to these pollutants^1^. Among them, endocrine-disrupting chemicals (EDCs) that may mimic and alter the function of hormones, have become a particular concern. Benzophenones (BPs) are one of the EDCs^2,3^ which are incautiously used as UV filters in personal care products (PCPs) and plastic packaging^4^. Benzophenones are also applied as flavouring ingredients, for the stabilization of odour, in sleeping tablets as well as in printing factories (IARC 2014)^5^. These compounds also naturally exist in several fruits and drinks like grapes, papaya and green tea^6,7^. BPs can be entered into the body by inhalation and ingestion, and through absorption on the skin. Recent studies have detected different BPs in water bodies and foods, and their metabolites in urine, blood and breast milk^8–10^. The main excretion path of the absorbed metabolites is via urine. Therefore, the level of exposure to the BPs can be determined by monitoring their metabolites in the urine^11^.

Some of the potential health impacts of exposure to BP derivatives include carcinogenicity, endocrine dysfunction, eczema, toxicity to the ecosystem, growth, reproduction system and organs^12,13^. According to the results of different studies and IARC reports, these chemicals are carcinogens to animals and potential carcinogens for humans (group B)^5^. 4-hydroxy benzophenone (4-OH-BP) and 2-hydroxy-4-methoxy benzophenone (BP-3) as derivatives of BP, with estrogenic activity and estrogen disruption properties, can accumulate in the blood, liver and kidneys through penetration from the skin and cause endometriosis and breast cancer^14–18^.

Over the last decades, the BPs containing products' widespread usage and the risks associated with them have attracted the attention of researchers. The most vulnerable group to these pollutants is pregnant women and their infants. BPs in a detectable concentration can cross the placenta and enter the mother's amniotic fluid and fetus's blood circulation potentially affecting the fetus's normal development process^19^. Many studies on the biological monitoring of BP metabolites have been performed in different countries, however, studies on pregnant women and their neonates are not extensive. It has been reported that BPs can alter the hormonal balance and interrupt the evolution of the reproductive system of the fetus^4,20,21^.

In China, monitoring the concentration of BP metabolites in pregnant women showed that increasing each logarithmic unit in 2,4-dihydroxy benzophenone (BP-1) and 4-OH-BP concentrations in the 1st trimester led to a 0.06 and 0.08 cm decrease in infant’s birth height, respectively. Reduced birth weight of newborns especially in girls was also associated with BP-1 and BP-3^19^. Research has shown that maternity exposure to BP-3 reduces the pregnancy duration and birth weight of girls, but conversely, increases male infants' head circumference and birth weight by up to 0.3 cm^22–25^. Furthermore, paternal preconception exposure and maternal exposure to BP-2 significantly increased the chance of a baby girl’s birth, while exposure to 4 -OH-BP during this period increased the chance of a baby boy’s birth^26^.

Considering the presence of BPs in a variety of substances and their adverse health effects on newborn infants and pregnant women, we monitored the most common and dangerous BP metabolites' urinary levels including BP-1, 4-OH-BP, BP-3 and 2,2′-dihydroxy-4-methoxy benzophenone (BP-8), in the first and third trimesters, the critical periods of pregnancy. In addition, we evaluated their association with birth outcomes such as birth length, weight and head, thoracic and arm circumference, anterior fontanelle diameter (AFD), infant sex, birth anomalies and corpulence index (CI). The association of urinary concentration of these metabolites with gestational age and the effects of gestational duration on infant's birth outcomes were evaluated comprehensively in our previous article^27^. In evaluating fetus growth, AFD is one of the most important parameters^28^. The average normal size of AFD at birth is 2.1 cm and usually, its large or small size indicates skull or systemic disorders in the body. The most common symptoms of large and protruding AFD are achondroplasia, congenital hypothyroidism, Down syndrome and rickets^28,29^.

Although a few studies have evaluated maternal exposure to other phenolic compounds such as parabens in Iran^30,31^, as far as the authors’ knowledge, this study was conducted for the first time in this country, one of the huge cosmetic consumer regions in the world. Furthermore, the impact of some metabolites such as BP-8 on birth outcomes has not been reported by now in the literature. Because very limited studies regarding BP-1 and 4-OH-BP effects on birth outcomes are available, identifying the factors affecting this outcome is very important. Therefore, the results of this study can be helpful for future research and also for healthcare authorities, especially in the case of assessing AFD abnormality which can be a symptom of certain diseases in newborn infants^28,29^.

## Materials and methods

### The population of the study

The participants of this study were a number of PERSIAN Cohort (Persian Prospective Epidemiological Research Studies in Iran) pregnant women of Isfahan residences, the third most populous city and the second industrial hub of Iran. Individuals without any underlying and chronic diseases, having the same address for more than 1 year, and being in the first trimester of pregnancy were included in the study. Hormone therapy or having any hormone-related illnesses and working in occupations such as cosmetics manufacturing, packaging and printing industries and hairdressers that increase the risk of high exposure to BPs, were considered exclusion criteria. In the present study, 170 eligible pregnant women were enrolled in 2019–2020. However, some of them (N = 4) due to miscarriage were excluded from the study before the sampling. Sampling was conducted during the routine pregnancy visits of 166 remaining participants. In the early morning, fasting spot urine samples were taken from the participants. All the participants signed a consent form describing the objectives of the study, voluntarily participating and the needed information in the questionnaire. Trained experts recorded the demographic, socio-economic and lifestyle characteristics of the study population through a direct interview. The participants' pregnancy weight was measured in three stages, the first and third trimesters and before giving birth, in the hospital with a calibrated instrument. However, the pre-pregnancy weight was recorded based on their declaration. The birth outcomes of newborns were measured using the standard and calibrated devices and recorded by experienced nurses. The birth data for 166 infants belonging to the studied women were recorded immediately after delivery. The protocol of the current study were approved by the Persian Cohort Ethics Committee (IR.MUI.REC.1394.1.354), and all the methods used in the study were performed in accordance with the relevant guidelines and regulations^32^.

### Chemicals and instruments

Analytical standards of BP-8, BP-1, BP-3 and 4-OH-BP; MSTFA and β-glucuronidase enzyme were provided from Sigma Aldridge (St. Louis, MO, USA). Analytical grade acetone, methanol and trichloromethane as dispersing and extraction solvents were purchased from Merck (Darmstadt, Germany). Stock solutions of the target compounds were prepared in methanol and stored in a dark vial at 4 °C until use. For preparing working standard solutions, the stock solution was diluted in synthetic urine and used for GC/MS calibration. An Agilent technology GC/ triple quadrupole MS was used for the quantification of the BP metabolites.

### Sample collection, preparation and analysis

Participants were asked to take a fasting urine sample by referring to the designated health centres in the early morning without changing their routine life patterns. The samples in the 1st and 3rd trimesters were taken within 10–14 weeks and 32–36 weeks of pregnancy, respectively. The urine samples were collected and stored at − 20 °C until analysis in polypropylene containers.

For extraction of the analytes, the dispersive liquid–liquid microextraction (DLLME) method was used which was explained in our previous study^27^. Briefly, 3–5 ml of the urine sample was added to 100 μL ammonium acetate in a falcon tube (pH = 6.8). ß-glucuronidase/arylsulfatase was used to complete enzymatic digestion. For extraction, trichloromethane and acetone were mixed and added to the digested sample to make a cloudy state. After centrifuging the solution, the organic phase dried with nitrogen gas blow-up, and then MSTFA was added and centrifuged for a complete mix. Eventually, 1 μL of the obtained solution was injected into the GC/MS^33–35^.

Separation was conducted by an HP-5 silica column with 3 m length, 0.25 mm I.D. and film thickness of 0.25 μm. Ultrapure helium was used as a carrier gas with a flow rate of 1 ml/min^−1^. Perfluorotributylamine was used to calibrate the mass spectrometer. To increase the accuracy and sensitivity, the selected ion monitoring (SIM) mode was set and mother ions of 193, 227, 285 and 343 m/z with the highest frequency were selected for quantification of 4-OH-BP, BP-3, BP-1 and BP-8, respectively. Initially, for separation of the metabolites, the column oven temperature was set at 90 °C for 2 min, then with an 8 °C/min temperature ramp raised to 290 °C, and at this temperature, it was kept for 5 min. Splitless was set for the injection mode and the temperature of the transfer line was kept at 290 °C.

### Calibration curve creation and analytical method validation

According to the literature, synthetic urine sample was used for calibration, due to the lack of human urine samples without any EDCs and matrix effects on calibration^36^. To create calibration curves, 0–100 µg/L of the four target benzophenone metabolites were spiked in the synthetic urine samples. The correlation coefficient (R^2^) of the calibration curve for BP-1, BP-3, BP-8 and 4-OH-BP were achieved at 0.996, 0.996, 0.993 and 0.993, respectively. For validation and reproducibility of the analytical method, quality control (QC) samples were used. A known amount of BP metabolites solution spiked into synthetic urine to achieve the target concentration of QC samples. All the procedures of the preparation method were applied for the spiked samples. Relative standard deviations (RSD) were calculated as an expression of the method's precision. Also, the accuracy of the method was investigated by the determination of the recovery percentage of known concentration of the target metabolites in synthetic urine samples. The average recovery percentage for the studied metabolites was 86.67%. However, we corrected our data with the correction factor of 1+ the recovery differences percentage achieved from spike samples measurements. Also, blank samples (synthetic urine without any BP metabolites) were used to investigate any possible contamination of samples by the experimental method. The limit of detection (LOD) and limit of quantification (LOQ) for each of the BP metabolites were calculated with 3 and 10 signal-to-noise ratios, respectively. LOD for, BP-1, BP-3, BP-8 and 4-OH-BP were 0.055, 0.045, 0.04 and 0.048 µg/L, respectively.

### Measurement of birth outcomes

Anthropometric indicators including weight, height, head, thoracic and arm circumference as well as AFD were recorded at birth, in the hospital by experienced nurses. Newborns' length was measured from crown to heel in a straight position, and they weighed completely naked. The largest diameter of the head, from the back of the head and above the nasal septum, was measured as the babies’ head circumference. Neonatal Apgar score was also assessed and recorded in the 1st and 5th minutes of birth. The infants were also examined by a paediatrician for birth defects, including any type of defect that can be diagnosed after birth.

### Covariates

The socio-demographic characteristics of mothers and infants and their classification are listed in detail in Table 1. The mothers’ BMI was calculated in the stages of pre-pregnancy, 1st and 3rd trimesters of pregnancy and classified according to the guidelines^37^. Information about gestational diseases such as diabetes and hypertension were achieved through testing in routine pregnancy visits. Gestational age was calculated at the time of delivery considering the date of delivery and the date of the last menstrual period (LMP)^31,38^.

**Table 1:** Characteristics of pregnant women and their infants.

	N (%) or mean ± SD
Maternal characteristics	
Maternal mean age (year)	29.94 ± 5.49
Maternal age (year)	
< 25	30 (18.52)
25–29	44 (27.16)
30–34	55 (33.95)
≥ 35	33 (20.37)
Pre-pregnancy BMI (kg/m^2^)	
< 18.5	14 (8.64)
18.5–23.9	61 (37.65)
24–29.9	71 (43.83)
≥ 30	16 (9.88)
BMI before delivery (kg/m^2^)	
< 18.5	0
18.5–23.9	12 (8)
24–29.9	64 (42.67)
≥ 30	74 (49.33)
Pregnancy weight gain (kg)	13.52 ± 6.22
Education	
≤ Diploma	100 (61.73)
Undergraduate	55 (33.95)
Postgraduate	7 (4.32)
Income (US Dollar)	
Low (< 300)	37 (23)
Moderate (300–500)	117 (72.70)
High (> 600)	7 (4.30)
Gestational diabetes	
Yes	10 (10.53)
No	85 (89.47)
Pregnancy blood pressure	
Yes	3 (3.16)
No	92 (96.84)
Passive smoking during first trimester	
Yes	39 (24.68)
No	119 (75.32)
Passive smoking during third trimester	
Yes	18 (22.22)
No	63 (77.78)
Pregnancy supplement	
Yes	155 (97.48)
No	4 (2.52)
Infant characteristics	
Sex	
Male	94 (56.63)
Female	72 (43.37)
Birth weight (g)	2961.68 ± 832.6
Birth length (cm)	47.25 ± 11.77
Head circumference (cm)	32.37 ± 8.44
Thoracic circumference (cm)	32.87 ± 1.5
Arm circumference (cm)	10.49 ± 0.79
Anomalies	3 (1.8)
Gestational age (week)	39 ± 2
Anterior fontanels diameter (cm)	2.2 ± 0.76
Apgar	9.89 ± 0.33
Corpulence index (g/cm^3^)	2.51 ± 0.34

### Statistical analysis

The linear regression models were used to examine the associations between BP metabolites and birth outcomes. The standardized regression model was used to evaluate the effect size of the metabolites on birth outcomes^39,40^. In addition, to determine the rate of changes in the birth outcomes per unit increase of BP metabolites, the unstandardized single-linear regression model was applied. For this purpose, the statistically significant variables in the standardized multiple-linear regression model were log-transformed and introduced to the single-linear regression model. The potential confounders including gestational age, weight gain during pregnancy, pre-pregnancy BMI, passive smoking, household monthly income and education were taken into account in the correlation analysis. All of the mentioned statistical analyses were performed once for the total infants and then for sex-stratified male and female infants, in both the first and third trimester of pregnancy.

The level of BP metabolites that were less than LOD was replaced with LOD values divided by a square root of two. Also, the creatinine level of the samples, due to the possible differences in urine samples' dilution, was analyzed using the Jaffe method with Hitachi 704 auto-analyzer. After analysis, the volume-based concentration (μg/L) of the metabolites divided by the creatinine concentration (g/L) of urine samples, so that, adjusted with creatinine levels (μg/g Cr). However, statistical analysis was performed for both creatinine-adjusted and unadjusted data. The SPSS software, V.26 (SPSS Inc., Chicago, IL), based on two-tailed tests, was used for the statistical analyses and a *p* value < 0.05 was set as statistical significance.

## Results and discussion

### The characteristics of the study population

In this study, the correlation between maternal exposure to the most common BP compounds and birth outcomes in a population in central Iran was investigated. Although Iran is ranked the second-largest cosmetic consumer in the Middle East^41^, and these substances contain BPs that affect human health especially pregnant women and their neonates, no human studies have been conducted on BPs biomonitoring and their health effects in Iran.

Table 1 represents the demographic characteristics of the study population. The average age of pregnant women was 29.94 ± 5.49 (17–43) years. Before pregnancy, 46.29% of them had lean or normal BMI, 43.83% were overweight, and 9.88% were in obese group. During pregnancy, the mothers' average weight gain was 13.52 ± 6.22 kg. |Majority of them (61.73%) had a diploma or primary education. About 71% of the participants had a moderate monthly income. The mean neonatal AFD was 2.2 ± 0.76 cm and their corpulence index was 2.51 ± 0.34 g/cm^3^.

### Concentrations of urinary BP metabolites

Table 2 shows the distribution of urinary BP metabolites concentration, based on urine volume (µg/L) and creatinine adjusted (µg/g Cr), in pregnant women in the 1st and 3rd trimesters of pregnancy. The highest concentrations of the metabolites were observed in the 1st trimester of pregnancy. The predominant compound with the highest concentration among other metabolites was BP-3, with a median concentration of 7.50 and 11.32 µg/g Cr, in the 1st and 3rd trimesters, respectively. These findings were in line with the results of previous research^19,42,43^. Considering that the study population has resided in a warm and dry region of Iran with a high UV index and intense sunlight, which enhances the consumption of sunscreen, as a health care product containing BP-3, these findings were acceptable. The lowest concentration in the current study was assigned to BP-8, which might be due to the short half-life of this metabolite and being a minor metabolite of BP-3^44^. 4-OH-BP showed a very significant decrease in the 3rd trimester (5.31 µg/g Cr) compared to the 1st trimester (13.43 µg/g Cr). This is a hydroxide metabolite of BP with a lower molecular weight compared to the other assessed metabolites. It can easily cross through the placenta and enter the fetal body. According to the literature, the transmission rate of 4-OH-BP from mother to fetus has been reported to be higher than other metabolites which supports our findings. Probably, this metabolite has a higher estrogenic activity even more than BP-3 which may explain the associations with fetal growth^8^. Thus, more attention is needed to regulate its use in cosmetic products and to monitor its exposure^44–46^.

**Table 2:** Distribution of urinary metabolites of benzophenone derivatives in the first and third trimester of pregnancy, volume base (µg/L) and creatinine adjusted (µg/g Cr).

Metabolite	Mean	SD	Min	Percentiles					Max
				5	25	50	75	95	
First trimester									
Volume base (µg/L)									
4-OH-BP	7.96	7.52	< LOD	0.24	2.1	5.75	11.7	22.26	46.2
BP-3	8.73	6.03	0.4	1.64	3.58	7.1	13.45	20.09	28
BP-1	5.35	4.75	0.3	1.04	2.2	4.05	7.8	11.9	42.9
BP-8	1.45	0.95	< LOD	0.2	0.2	1.7	1.9	2.67	6.6
Creatinine adjusted (µg/g)									
4-OH-BP	15.01	35.42	< LOD	0.28	2.24	6.62	13.43	51.31	377.78
BP-3	14.65	22.31	0.41	1.22	3.84	7.5	17.53	58.04	205.75
BP-1	9.66	17.43	0.31	0.67	2.22	4.39	10.24	39.6	151.77
BP-8	2.53	3.93	< LOD	0.13	0.58	1.32	2.3	11.6	23.12
Third trimester									
Volume base (µg/L)									
4-OH-BP	3.15	2.9	0.1	0.15	1.2	2.9	4.9	6.9	7.4
BP-3	17.32	17.4	5.4	5.8	11.6	17.4	23.55	29.2	31.2
BP-1	10.03	10.1	3.3	3.5	6.8	10.1	13.5	16.8	17.9
BP-8	1.03	1	0.1	0.45	0.8	1	1.1	1.9	1.9
Creatinine adjusted (µg/g)									
4-OH-BP	3.52	3.15	0.08	0.13	1.13	3.15	5.31	9.36	9.6
BP-3	19.66	16.98	4.73	5.25	10.99	16.98	26.06	45.65	49.47
BP-1	11.37	9.95	2.86	3.19	6.44	9.95	15.02	26.02	27.95
BP-8	1.2	1.04	0.04	0.28	0.76	1.04	1.56	2.86	2.95

**Figure 1:**
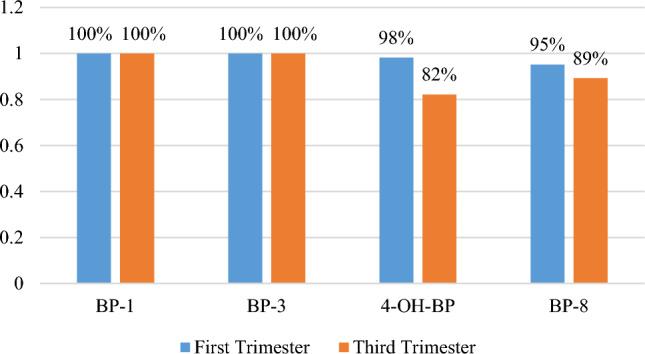
The detection rate of benzophenone derivatives in the first and third trimester of pregnancy.

Similar to 4-OH-BP, BP-8, BP-1 and BP-3 can be easily transferred from mother to fetus due to their low molecular weight. Studies have shown that the median concentration of these metabolites in cord serum was higher compared to pregnancy serum. This reveals that the fetus is more likely than the mother to be exposed to these compounds, which may have harmful impacts on the health and normal development of the fetus during pregnancy^19,46^. In the current study, all metabolites were detected in the samples with a high percentage. BP-1 and BP-3 were detected in 100% of the samples in the first and third trimesters. 4-OH-BP in the first and third trimesters were identified in 98% and 82%, and BP-8 in 95% and 89% of the samples, respectively (Fig. 1). Our achieved detection rates were much higher than that in the study conducted in China. The detection rates of their study were 91.7% and 89.3% for BP-1, 76.5%, 67.4% for BP-3, 86.7% and 86.9% for 4-OH-BP in the first and third trimester, respectively^19^. This difference probably is due to the very low LOD (BP-3: 0.045, BP-1: 0.055, 4-OH-BP: 0.048 µg/L) of these metabolites in our study compared with the presented LOD (BP-3: 0.2, BP-1: 0.1, 4-OH-BP: 0.1 µg/L) in that study^47^. Another study from china detected very lower rates of these three metabolites in both the first and third trimester of pregnancy^48^. The detection rate of BP-8 in the present study in the first and third trimesters was 95% and 89%, respectively. However, in most studies on pregnant women, the BP-8 has not been analyzed, or it was detected at a very low rate, about 13%^46^ and 2%^42^. These contradictory results can be attributed to the low LOD of this compound in the present study or the high median concentration of BP-8 (1.1–1.9 µg/L) in the first and third trimester of pregnancy probably due to the use of high BP-8-containing compounds. While in the mentioned studies the median concentrations were below 0.001 µg/L. Nevertheless, in our study, all the metabolites' detection rates are in accordance with the results of a study that evaluated BPs urinary concentrations in Chinese and American adults and children^45^.

**Figure 2:**
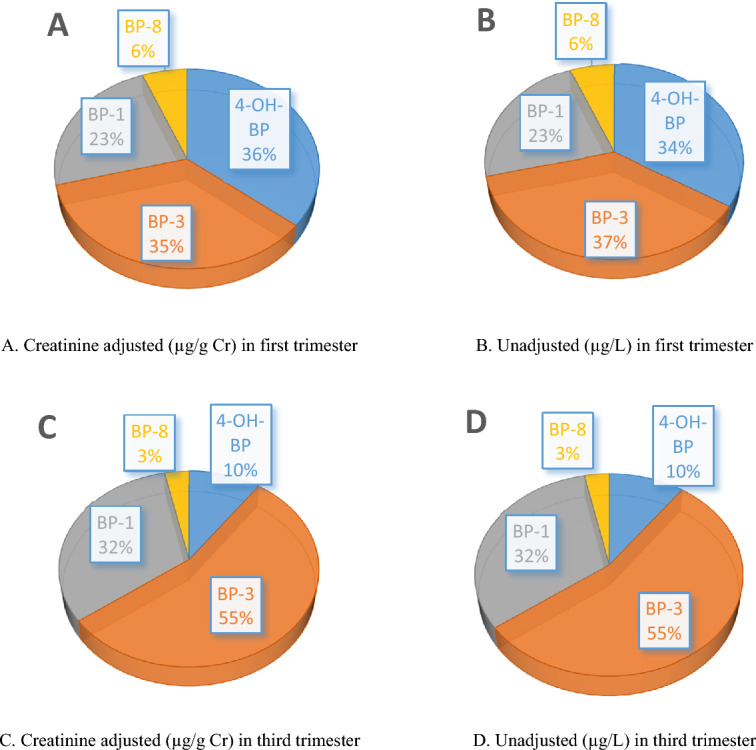
Composition profiles of BP-derivatives in the urine samples of Iranian pregnant women.

The composition profiles of BP derivatives in urine in the first and third trimesters are illustrated in Fig. 2. No changes in the ratio of metabolites in volume-based and creatinine-adjusted states were observed which indicates that the confounder factors have not noticeably influenced their concentration in the urine samples. The most common compound in both trimesters was BP-3, followed by 4-OH-BP in the first trimester and BP-1 in the third trimester. It was noteworthy that the BP-3 ratio increased from 35% in the 1st trimester to 55% in the 3rd trimester. This seems to be due to the high sequential use of the products containing BP-3 for a long time.

Table 3 shows a comparison of the median concentrations of maternal BP metabolites in this study with those reported in other countries. According to this table, BP-3 is the only metabolite that has been analyzed in the other studies, whereas we analyzed four common metabolites. The highest concentrations of BP-3 belonged to the United States, followed by Puerto Rico and Iran. Americans with the same age, sex and amount of body fat with Asians, tend to have a consistently higher BMI by about 2–3 kg/m^2^, partly because of the differences in body build and muscularity^49^. Obesity and overweight cause a delay in the metabolism of BPs, leading to an increase in their levels in blood and urine^50^. In addition, these areas have very hot summers where people need to use a lot of lotions and sunscreens to protect themselves from the sun's UV rays. However, in Iran, due to cultural restrictions and religious beliefs, outdoor swimming pools or beach areas for swimming and sunbathing, which increase the need to use sunscreen for the whole body, are not used by women, so a small part of the skin comes in contact with BP-3 containing products. This could be proof of the lower concentration of BP-3 in our study compared to the US and Puerto Rico islands. Nevertheless, the concentrations of other metabolites (4-OH-BP, BP-1, BP-8) in our study were significantly higher than those reported in other countries. This can be attributed to the high use of cosmetics in this country, many of which are probably of low quality due to economic problems or the lack of strict rules for the production and use of these chemicals.

**Table 3:** Comparison of median concentration of BP metabolites in maternal urinary samples in Iran and those reported in other countries (µg/L).

Country/city	Number	Sampling period (year)	BP-1	BP-3	BP-8	4-OH-BP	References
USA/New York	404	1998–2002	NA	7.5	NA	NA	^23^
USA/Massachusetts	213	2005–2016	NA	137.6^a^	NA	NA	^51^
USA/Boston	476	2006–2008	NA	46.5^a^	NA	NA	^52^
USA/ New York	71	2005–2008	NA	53.5	NA	NA	^53^
China/Wuhan	644	2014–2015	0.31^a^	0.65^a^	NA	0.13^a^	^54^
China/Qingyuan	97	2016–2017	2.85	0.42	< 0.01	0.67	^46^
China/Nanihng	101	2009–2014	NA	0.08	NA	NA	^55^
China/Wuhan	847	2014–2015	0.30	0.59	NA	0.14	^19^
France	191	2002–2006	NA	1.7	NA	NA	^22^
Puerto Rico	105	2010–2012	NA	31.3	NA	NA	^56^
Iran	166	2019–2020	4.05	7.1	1.7	5.75	Current study

^a^Specific gravity-corrected concentrations of BPs in urine were reported in these studies, therefore we excluded these studies for comparison.

### Effects of urinary BP metabolites on birth outcomes of total infants

Table 4 represents the relationship between creatinine-adjusted concentrations of BP metabolites and neonatal birth outcomes, and the changes (β with 95% confidence interval) in the SD of the outcomes’ values per 1-SD increase in the metabolites’ concentration in the first and third trimester of pregnancy. According to the table, in the first trimester, 4-OH-BP showed a significant inverse association with AFD (β: − 0.490; 95% CI − 0.976, − 0.004). Also, this metabolite represented a positive association with neonatal head circumference (β: 0.447, 95% CI − 0.165, 1.058) and arm circumference (β: 0.573, 95% CI − 0.081, 1.226). The higher β value indicates a stronger effect size, thus, the effect of 4-OH-BP on infants’ arm circumference was quite stronger than that for head circumference. Table 5 shows the changes in significantly correlated birth outcomes per 1 log unit increase of the BP metabolites. Unstandardized regression coefficient (B) reveals that one log-unit increase of 4-OH-BP was correlated with a decrease of 0.034 cm (95% CI − 0.380, 0.312) in AFD and an increase of 0.091 cm (95% CI − 0.067, 0.086) in head and 0.317 cm (95% CI 0.027, 0.607) in arm circumferences in the first trimester.

**Table 4:** Standardized regression coefficients [β (95% CI)] for associations of creatinine adjusted concentrations (µg/g Cr) of BP derivatives in first and third trimesters with birth outcomes in total infants.

Metabolite	Infant Sex	Birth weight	Birth length	Head circumference	Thoracic circumference	Arm circumference	Fontanels diameter	Birth anomalies	Corpulence index
4-OH-BP									
First trimester	0.109 (− 0.353,0.571)	− 1.721 (− 5.078, 1.637)	2.232 (− 2.297, 6.762)	0.447 (− 0.165, 1.058)*	− 0.332 (− 1.320, 0.657)	0.573 (− 0.081, 1.226)*	− 0.490 (− 0.976, − 0.004)**	− 0.043 (− 0.533, 0.447)	1.89 (− 2.236, 6.015)
Third trimester	0.192 (− 3.272,2.117)	− 0.192 (− 2.651, 2.524)	− 0.19 (− 1.178, 3.364)	− 0.214 (− 0.595, 3.990)	− 0.364 (− 2.096, 7.107)	− 0.316 (− 1.151, 3.994)	0.126 (− 1.495, 0.796)	0.009 (− 0.171, 0.040)	− 0.117 (− 2.032, 0.685)
BP-3									
First trimester	0.042 (− 0.435,0.520)	− 2.111 (− 5.580, 1.359)	3.25 (− 1.430, 7.931)	0.216 (− 0.416, 0.847)	− 0.27 (− 1.291, 0.752)	0.498 (− 0.177, 1.173)*	− 0.141 (− 0.674, 0.393)	− 0.147 (− 0.653, 0.359)	2.711 (− 1.551, 6.974)
Third trimester	0.159 (− 1.285,0.404)	− 0.035 (− 0.536, 0.407)	− 0.12 (− 0.439, 0.601)	− 0.014 (− 0.166, 0.183)	− 0.212 (− 3.085, 3.397)	− 0.316 (− 5.711, 0.791)	0.172 (− 2.274, 1.046)	0.118 (− 1.048, 0.842)	0.039 (− 0.096, 0.087)
BP-1									
First trimester	0.014 (− 0.512,0.540)	− 0.289 (− 4.115, 3.536)	0.619 (− 4.542, 5.780)	− 0.042 (− 0.739, 0.655)	− 0.224 (− 1.351,0.902)	0.043 (− 0.701, 0.787)	− 0.03 (− 0.618,0.558)	− 0.049 (− 0.607, 0.509)	0.19 (− 4.510,4.891)
Third trimester	0.151 (− 2.204,2.913)	− 0.046 (− 0.719, 0.613)	− 0.132 (− 0.697, 0.276)	− 0.024 (− 0.313, 0.396)	− 0.212 (− 3.551, 0.703)	− 0.316 (− 4.601, 4.370)	0.172 (− 2.370, 2.134)	0.118 (− 1.166, 1.443)	0.042 (− 0.818, 0.789)
BP-8									
First trimester	0.195 (− 0.255,0.644)	− 0.877 (− 4.146, 2.392)	1.648 (− 2.762, 6.058)	− 0.188 (− 0.783, 0.407)	0.356 (− 0.607, 1.318)	0.309 (− 0.327, 0.945)	0.117 (− 0.386, 0.620)	0.004 (− 0.473, 0.480)	1.309 (− 2.707, 5.326)
Third trimester	0.025 (− 0.484,0.496)	0.043 (− 0.198, 0.159)	0.067 (− 1.089, 0.635)	− 0.13 (− 2.404, 1.671)	0.348 (− 6.071, 5.122)	0.949 (− 0.456, 2.147)**	− 0.077 (− 0.835, 1.318)	0.22 (− 4.093, 0.872)	− 0.06 (− 0.338, 1.138)

4-hydroxy benzophenone (4-OH-BP), 2-hydroxy-4-methoxy benzophenone (BP-3), 2,4-dihydroxy benzophenone (BP-1), and 2,2′-dihydroxy-4-methoxy benzophenone (BP-8).

*0.05 < *p* value < 0.1, **p value < 0.05, Model adjusted for gestational age, weight gain during pregnancy, pre-pregnancy BMI, passive smoking, education and household monthly income. Values indicate the change in each outcome (in SD units) predicted by a 1-SD change in the metabolites.

**Table 5:** Unstandardized regression coefficients [B (95% CI)] for changes in significantly associated birth outcomes per log-transformed unit of BP metabolites in first and Third trimesters.

Metabolite	Infants' group	Birth outcome-Trimester	[B (95% CI)]
4-OH-BP	Total infants	Head circumference (cm)-1st	0.091 (− 0.067, 0.086)
		Arm circumference (cm)-1st	0.317 (0.027, 0.607)
		AFD (cm)-1st	− 0.034 (− 0.380, 0.312)
	Boys	Head circumference (cm)-1st	0.828 (− 1.510, 2.946)
		Weight (g)-1st	− 49.693 (− 116.635, − 11.537)
	Girls	Head circumference (cm)-3rd	− 3.301 (− 1.284, 2.149)
		Weight (g)-3rd	− 10.455 (− 133.418, 110.457)
		AFD (cm)-3rd	0.141 (− 0.850, 1.112)
		Thoracic circumference (cm)-3rd	− 0.888 (− 3.184, 2.374)
		Arm circumference (cm)-3rd	− 0.063 (− 1.816, 0.957)
BP-3	Total infants	Arm circumference (cm)-1st	0.374 (− 0.046, 0.522)
	Boys	Head circumference (cm)-1st	0.914 (0.001, 2.580)
		Weight (g)-1st	− 12.729 (− 48.134, 14.248)
		Corpulence index-1st	0.012 (− 0.140, 1.184)
	Girls	Arm circumference (cm)-3rd	0.197 (− 1.207, 1.549)
		AFD (cm)-3rd	− 0.250 (− 0.949, 0.380)
BP-1	Total infants	–	–
	Boys	Head circumference (cm)-1st	0.712 (− 1.290, 1.173)
		Weight (g)-1st	− 55.575 (− 100.014, 227.219)
		Corpulence index-1st	0.039 (− 0.780, 0.584)
	Girls	–	–
BP-8	Total infants	Arm circumference (cm)-3rd	0.487 (0.087, 0.948)
	Boys	Thoracic circumference (cm)-3rd	0.722 (− 1.740, 1.921)
		AFD (cm)-3rd	0.220 (− 0.625, 1.417)
	Girls	–	–

*1st* first trimester, *3rd* third trimester.

Despite the great importance of AFD on neonatal health, no study examined the effect of benzophenone exposure during pregnancy on offspring AFD. However, a study that evaluated the size of neonatal fontanelle at birth, demonstrated that for each unit increase in neonatal head circumference, the size of AFD increases up to 52%^29^. Therefore, 4-OH-BP possibly not only reduces the size of the neonatal AFD but also increases the risk of having an abnormal fontanelle size by increasing the size of head circumference. According to Table 4, BP-3 had also a positive effect on the size of the neonatal arm circumference in the first trimester. Also, BP-8 showed a positive association with arm circumference in total neonates in the third trimester (β: 0.949, 95% CI − 0.456, 2.147). However, there was no significant association between the other metabolites and birth outcomes. Apart from that, in the first trimester, there was no significant association between both BP-3 and BP-1 with birth weight, which was in complete agreement with the study of 182 Danish mothers^8^. According to the previous reports, the correlations of maternal exposure to EDCs with infants' birth outcomes could be sex-dependent^22,23,51^. Therefore, to determine the possible sex-specific effects of the BP derivatives additional stratification of the analysis was conducted by neonatal gender.

### Effects of urinary BP metabolites on birth outcomes by infants’ sex

Table 6 describes the correlation between the concentrations of BP metabolites and birth outcomes in boys in the first and third trimesters. The head circumference of male neonates was significantly affected (β: 0.990, 95% CI − 0.174, 2.175) by 4-OH-BP urinary concentration measured in the first trimester. Furthermore, 4-OH-BP, BP-1 and BP-3 were all inversely related to the weight of male infants in the first trimester. In addition, BP-3 and BP-1 represented a noticeable direct association with head circumference and corpulence index of boys in the first trimester with the nearly same effect sizes. In a Chinese neonatal study, there was no significant correlation between BP-1 and BP-3 and male infants’ weight. However, in their study in the first trimester, a significant correlation (β: − 0.1, 95% CI − 0.17, − 0.03) was found between BP-1 and the length of male infants^19^, of which we found no association. In the EDEN cohort study in France, per 1-unit increase in ln-transformed BP-3 concentration, 26 g of male birth weight and 0.1 cm of head circumference were increased, while in our study, a log-unit increase of BP-3 metabolite decreased by 12.8 g of male birth weight but increased 0.9 cm of head circumference (Table 4)^22^. However, Messerlian et.al found no relationship between BP-3 and male head circumference^51^. Different behaviour of BP-3 may be attributed to its anti-androgenic and estrogenic activities and the level of exposure^57^. The findings of similar studies that evaluated the impacts of pregnancy exposure to BPs on male infants in France confirmed the results of the EDEN cohort study^22,58^. Whereas, researchers in children's Environmental Health Study have not achieved a significant association between BP-3 in the third trimester of pregnancy with weight, length and head circumference of the neonates^23^. We achieved a direct association between BP-8 in the 3rd trimester with the size of AFD (β: 0.433, 95% CI 0.423, 1.00) and thoracic circumference (β: 0.821, 95% CI 0.263, 1.05) in male infants, with twice stronger effect size on thoracic circumference than AFD. BP-8 due to the shorter half-life and difficulty in detection was neglected among researchers and we found no study by now that investigate the effects of BP-8 on these birth outcomes.

**Table 6:** Standardized regression coefficients [β (95% CI)] for associations of creatinine adjusted concentrations (µg/g Cr) of benzophenone derivatives in first and third trimesters with birth outcomes in boys.

Metabolite	Birth weight	Birth length	Head circumference	Thoracic circumference	Arm circumference	Fontanels diameter	Birth anomalies	Corpulence index
4-OH-BP								
First trimester	− 0.997 (− 2.68, 1.689)*	5.043 (− 4.962, 15.048)	0.990 (− 0.174, 2.175)**	0.547 (− 2.853, 3.948)	0.859 (− 0.826, 2.545)	− 0.481 (− 1.762, 0.800)	− 0.881 (− 2.301, 0.540)	4.503 (− 3.975, 12.980)
Third trimester	0.281 (− 1.516, 4.066)	− 0.11 (− 0.852, 1.391)	0.135 (− 0.401, 0.788)	0.154 (− 2.139, 1.460)	− 0.866 (− 7.370, 8.885)	0.141 (− 0.439, 1.152)	0.039 (− 0.194, 0.235)	0.154 (− 1.089, 1.515)
BP-3								
First trimester	− 0.995 (− 2.696, 1.696)*	5.578 (− 4.193, 15.350)	0.931 (− 0.428, 2.291)*	0.755 (− 2.567, 4.076)	0.758 (− 0.888, 2.405)	− 0.264 (− 1.515, 0.987)	− 1.002 (− 2.390, 0.385)	0.999 (− 0.705, 2.705)*
Third trimester	0.227 (− 3.078, 3.414)	− 0.255 (− 2.160, 4.299)	0.197 (− 0.670, 3.789)	− 0.154 (− 0.129, 1.179)	− 0.866 (− 7.950, 11.414)	− 0.035 (− 0.274, 0.109)	0.072 (− 0.265, 0.861)	0.314 (− 3.266, 0.841)
BP-1								
First trimester	− 0.994 (− 2.693, 1.693)*	5.577 (− 4.018, 15.172)	0.906 (− 0.429, 2.241)*	0.744 (− 2.517, 4.005)	0.741 (− 0.875, 2.358)	− 0.252 (− 1.480, 0.977)	− 1.002 (− 2.364, 0.361)	0.998 (− 0.680, 2.680)*
Third trimester	0.227 (− 1.279, 1.794 )	− 0.255 (− 3.733, 1.234)	0.197 (− 1.356, 2.676)	− 0.154 (− 1.283, 2.173)	− 0.866 (− 13.683, 12.635)	0.107 (− 1.393, 1.347)	0.072 (− 0.651, 1.428)	0.314 (− 0.809, 2.296)
BP-8								
First trimester	− 2.275 (− 8.616, 4.065)	4.127 (− 5.747, 14.002)	0.488 (− 0.886, 1.862)	0.676 (− 2.681, 4.032)	0.499 (− 1.165, 2.163)	0.062 (− 1.202, 1.327)	− 0.773 (− 2.175, 0.629)	3.538 (− 4.830, 11.905)
Third trimester	0.31 (− 1.866, 4.309)	− 0.026 (− 0.219, 0.488)	0.282 (− 3.364, 2.403)	0.821 (0.263, 1.05)*	0.866 (− 6.036, 5.369)	0.433 (0.423, 1.00)**	0.161 (− 0.482, 2.128)	0.273 (− 3.930, 5.196)

4-hydroxy benzophenone (4-OH-BP), 2-hydroxy-4-methoxy benzophenone (BP-3), 2,4-dihydroxy benzophenone (BP-1), and 2,2′-dihydroxy-4-methoxy benzophenone (BP-8).

*0.05 < *p* value < 0.1, **p value < 0.05, Model adjusted for gestational age, weight gain during pregnancy, pre-pregnancy BMI, passive smoking, education and household monthly income. Values indicate the change in each outcome (in SD units) predicted by a 1-SD change in the metabolites.

The impacts of urinary BP derivatives levels, in the first and third trimesters of pregnancy on female infants, are shown in Table 7. In the first trimester, no significant association existed, however, in the 3rd trimester, 4-OH-BP represented a very significant inverse relationship with the weight of female infants (β: − 0.75, 95% CI − 0.905, 0.621). In addition, BP-3 demonstrated a very strong correlation with infant’s AFD (β: − 0.586, 95% CI − 0.993, − 0.576). Long et.al reported that BP-3 and BP-1 reduced the female's birth weight and length in the 3rd trimester, but 4-OH-BP had no effect on these outcomes, which contradicts the results of current studies^19^. Also, the study of American mothers in the third trimester showed that BP-3 reduced the birth weight of females and, conversely, increased the birth weight of males^23^. In our study, according to the unstandardized regression coefficient (B), BP-1 had the highest impact on birth weight among the other birth outcomes, decreasing male birth weight by 55.58 g (− 100.014, 227.219) per a log unit increase of BP-1, maybe due to its high estrogenic activity^48,59^. In addition, a 1-unit increase in log-transformed 4-OH-BP in the third trimester led to a 10.46 g birth weight decrease in female infants and that in the first trimester led to a 49.69 g decrease in male birth weight (Table 5). Within the metabolites, 4-OH-BP showed significant correlations with most of the studied parameters of birth outcomes in all the infant groups in both trimesters. This is probably due to the higher proportion of this compound in the urine of pregnant women and the higher transformation rate in the fetus due to the lower molecular weight of this metabolite compared to the others^45,46^.

**Table 7:** Standardized regression coefficients [β(95% CI)] for associations of creatinine adjusted concentrations (µg/g Cr) of benzophenone derivatives in first and third trimesters with birth outcomes in girls.

Metabolite	Birth weight	Birth length	Head circumference	Thoracic circumference	Arm circumference	Fontanels diameter	Birth anomalies	Corpulence index
4-OH-BP								
First trimester	− 5.333 (− 23.017, 12.350)	6.638 (− 14.166, 27.442)	0.123 (− 2.929, 3.174)	− 0.293 (− 6.373, 5.788)	0.654 (− 2.757, 4.065)	− 0.091 (− 3.271, 3.088)	− 0.721 (− 3.471, 2.029)	5.996 (− 12.376, 24.369)
Third trimester	− 0.75 (− 0.905, 0.621)**	− 0.348 (− 3.500, 1.501)	− 0.518 (− 2.912, 4.026)*	− 0.963 (− 2.831, 2.552)*	− 0.368 (− 0.783, 0.035)*	0.416 (− 0.1, 0.976)*	0.292 (− 1.252, 4.621)	− 0.318 (− 4.668, 3.427)
BP-3								
First trimester	− 5.64 (− 21.385, 10.105)	6.937 (11.586, 25.460)	0.147 (− 2.570, 2.864)	− 0.839 (− 6.252, 4.575)	0.561 (− 2.476, 3.598)	− 0.131 (− 2.962, 2.700)	− 0.81 (− 3.258, 1.638)	6.057 (− 10.301, 22.415)
Third trimester	0.035 (− 0.149, 0.618)	0.104 (− 0.867, 0.830)	0.059 (− 0.878, 0.198)	− 0.089 (− 1.758, 1.491)	0.492 (− 0.09, − 0.777)*	− 0.586 (− 0.993,− 0.576)**	0.325 (− 3.192, 1.845)	− 0.176 (− 1.647, 1.966)
BP-1								
First trimester	0.972 (− 16.257, 18.201)	− 1.267 (− 21.536, 19.003)	− 0.235 (− 3.208, 2.738)	− 0.451 (− 6.375, 5.473)	− 0.235 (− 3.558, 3.089)	− 0.032 (− 3.130, 3.066)	− 0.345 (− 3.024, 2.334)	− 1.333 (− 19.233, 16.568)
Third trimester	0.025 (− 0.115, 0.066)	0.1 (− 0.834, 0.781)	0.05 (− 0.496, 0.463)	− 0.097 (− 0.324, 1.400)	0.023 (− 0.215, 0.310)	− 0.347 (− 6.268, 6.907)	0.328 (− 6.187, 3.891)	− 0.183 (− 3.260, 3.627)
BP-8								
First trimester	− 2.244 (− 16.331, 11.843)	2.889 (− 13.683, 19.462)	− 0.018 (− 2.449, 2.413)	− 1.434 (− 6.278, 3.410)	0.149 2.568, 2.866)	− 0.204 (− 2.736, 2.329)	− 0.439 (− 2.630, 1.751)	2.123 (− 12.512, 16.759)
Third trimester	− 0.175 (− 3.303, 3.451)	0.047 (− 0.939, 0.461)	− 0.344 (− 5.159, 0.765)	0.269 (− 1.794, 1.384)	0.214 3.387, 2.932)	− 0.029 (− 0.357, 0.358)	0.237 (− 2.575, 4.425)	0.106 (− 0.576, 1.519)

Abbreviations: 4-hydroxy benzophenone (4-OH-BP), 2-hydroxy-4-methoxy benzophenone (BP-3), 2,4-dihydroxy benzophenone (BP-1), and 2,2′-dihydroxy-4-methoxy benzophenone (BP-8).

*0.05 < *p* value < 0.1, **p value < 0.05, Model adjusted for gestational age, weight gain during pregnancy, pre-pregnancy BMI, passive smoking, education and household monthly income. Values indicate the change in each outcome (in SD units) predicted by a 1-SD change in the metabolites.

In the current study, there was no significant association between the sex of infants and the studied metabolites. However, some studies stated that BP-3 in the growing fetus targets sex hormones and interferes with estrogen and progesterone level^24,60^. In the study of Korean mothers, a urinary level of 4-OH-BP was strongly associated with an excess birth rate of male infants^26^. Furthermore, in the current study corpulence index, thoracic and arm circumference variables showed significant correlations. However, the effect of the metabolites on arm circumferences was clearer than the others. As far as we know by now, there is no study on BPs exposure associated with these birth outcomes unless the Boston cohort study. According to their study, changes in the interquartile range (IQR) of BP-3 were correlated with a 0.26 decrease and 0.36 increase of standard deviation in the abdominal circumference of male and female infants, respectively^52^. We did not find any significant association between the BP derivatives and birth defects; this was probably due to the low percentage of neonate birth abnormalities in the study population.

Generally, the number of studies that examined maternal exposure to benzophenones and their effects on offspring outcomes is very limited. Usually, the conducted studies, have considered only the routine outcomes of birth, such as weight, height, and in some cases, head circumference. In addition, mostly the association of BP-3 with birth outcomes has been evaluated and other metabolites have not been investigated in a wide range. However, we tried to fill this gap by assessing the correlation of BP metabolites especially those suspected to have high estrogenic activity with a wide range of neonatal birth outcomes, which is the strong point of the study. In this regard, the results of this study can pave the way for further studies concerning the birth outcome of maternal exposure to benzophenones. Despite the many strengths of this study, the main limitation of the study was its coincidence with the Covid-19 pandemic, which caused many problems in sampling due to the high sensitivity of the study population. Given the broad pathways of exposure to BPs, including food and PCP products usage behaviour, it was impossible to select individuals without exposure to these chemicals as a control group. Therefore, the study was designed as a cross-sectional and the participant's urinary concentration of the BP metabolites were compared to each other, based on the level of exposure. This may be considered as another limitation of this study, because cross-sectional study cannot show causal relationships properly. However, this study is part of a cohort study which may cover this limitation and provide very useful information for the cohort study.

## Conclusion

This study evaluated exposure to benzophenone compounds during pregnancy and their possible health effects on birth outcomes including birth defects, arm circumference, corpulence index and anterior fontanelle diameter (AFD) along with other routine outcomes at birth. To the best of our knowledge, this subject has not been already studied in Iran, especially in the susceptible population such as pregnant women. We found very noticeable correlations within BP derivatives in both boys and girls. In total infants, 4-OH-BP in the first trimester showed a very significant correlation with AFD. In the third trimester, BP-8 was strongly associated with arm circumferences. The sex-specific analysis represented that 4-OH-BP in the first trimester and BP-8 in the third trimester had a considerable impact on boys’ head circumferences and AFD, respectively. However, 4-OH-BP, BP-3 and BP-1 had a negative correlation with boys’ birth weight in the first trimester. Within female neonates, in the first trimester no significant association was found, whereas, in the third trimester, the impacts of BP derivatives were more pronounced in girls with a significant converse correlation of 4-OH-BP with birth weight, and BP-3 and BP-1 with AFD. Our findings showed that all evaluated BP derivatives can affect fetus normal growth in the both early and late stages of pregnancy. However, due to the limited studies on this subject, further investigations are necessary to approve the results, especially regarding the scarcely evaluated birth outcomes.

## Data Availability

The supporting data are available from the corresponding authors upon reasonable request.

## References

[1] Jiménez-Díaz I (2016). Urinary levels of bisphenol A, benzophenones and parabens in Tunisian women: A pilot study. Sci. Total Environ..

[2] Darbre PD (2019). The history of endocrine-disrupting chemicals. Curr. Opin. Endocr. Metab. Res..

[3] Yang J, Shi S, Xu F, Nie J (2013). Synthesis and photopolymerization kinetics of benzophenone sesamol one-component photoinitiator. Photochem. Photobiol. Sci..

[4] Ghazipura M, McGowan R, Arslan A, Hossain T (2017). Exposure to benzophenone-3 and reproductive toxicity: A systematic review of human and animal studies. Reprod. Toxicol..

[5] International Agency for Research on Cancer (2012). Agents classified by the IARC monographs. Igarss 2014.

[6] Hazardous substances data bank. Ethyl silicate Last... - Google Scholar. https://scholar.google.com/scholar.

[7] Heurung AR, Raju SI, Warshaw EM (2014). Benzophenones. Dermatitis.

[8] Krause M (2018). Presence of benzophenones commonly used as UV filters and absorbers in paired maternal and fetal samples. Environ. Int..

[9] Delgado DM (2017). Determination of UV Filters in Human Breast Milk Using Turbulent Flow Chromatography and Babies’ Daily Intake Estimation Globaqua View Project SEA-on-a-CHIP View Project.

[10] Careghini A, Mastorgio AF, Saponaro S, Sezenna E (2015). Bisphenol A, nonylphenols, benzophenones, and benzotriazoles in soils, groundwater, surface water, sediments, and food: A review. Environ. Sci. Pollut. Res..

[11] Kawaguchi M (2009). Miniaturized hollow fiber assisted liquid-phase microextraction and gas chromatography-mass spectrometry for determination of benzophenone and derivates in human urine sample. J. Chromatogr. B Anal. Technol. Biomed. Life Sci..

[12] Kerdivel G (2013). Estrogenic potency of benzophenone UV filters in breast cancer cells: Proliferative and transcriptional activity substantiated by docking analysis. PLoS ONE.

[13] Li CC, Chen YT, Lin YT, Sie SF, Chen-Yang YW (2014). Mesoporous silica aerogel as a drug carrier for the enhancement of the sunscreen ability of benzophenone-3. Colloids Surf. B Biointerfaces.

[14] Kim S, Choi K (2014). Occurrences, toxicities, and ecological risks of benzophenone-3, a common component of organic sunscreen products: A mini-review. Environ. Int..

[15] Barone AN, Hayes CE, Kerr JJ, Lee RC, Flaherty DB (2019). Acute toxicity testing of TiO2-based vs. oxybenzone-based sunscreens on clownfish (*Amphiprion ocellaris*). Environ. Sci. Pollut. Res..

[16] Kunisue T (2012). Urinary concentrations of benzophenone-type UV filters in U.S. women and their association with endometriosis. Environ. Sci. Technol..

[17] Smarr MM, Kannan K, Buck Louis GM (2016). Endocrine disrupting chemicals and endometriosis. Fertil. Steril..

[18] Karthikraj R, Kannan K, Salvador A, Chisvert A (2018). Human biomonitoring of select ingredients in cosmetics. Analysis of Cosmetic Products.

[19] Long J (2019). Maternal urinary benzophenones and infant birth size: Identifying critical windows of exposure. Chemosphere.

[20] Nakamura N (2015). Effects of maternal and lactational exposure to 2-hydroxy-4-methoxybenzone on development and reproductive organs in male and female rat offspring. Birth Defects Res. Part B Dev. Reprod. Toxicol..

[21] Balázs A (2016). Hormonal activity, cytotoxicity and developmental toxicity of UV filters. Ecotoxicol. Environ. Saf..

[22] Philippat C (2012). Exposure to phthalates and phenols during pregnancy and offspring size at birth. Environ. Health Perspect..

[23] Wolff MS (2008). Prenatal phenol and phthalate exposures and birth outcomes. Environ. Health Perspect..

[24] Tang R (2013). Associations of prenatal exposure to phenols with birth outcomes. Environ. Pollut..

[25] Philippat C (2019). Prenatal exposure to select phthalates and phenols and associations with fetal and placental weight among male births in the EDEN Cohort (France). Environ. Health Perspect..

[26] Bae J, Kim S, Kannan K, Buck Louis GM (2016). Couples’ urinary concentrations of benzophenone-type ultraviolet filters and the secondary sex ratio. Sci. Total Environ..

[27] Teiri H (2022). The association of prenatal exposure to benzophenones with gestational age and offspring size at birth. Environ. Sci. Pollut. Res..

[28] Kiesler J, Ricer R (2003). The abnormal fontanel. Am. Fam. Physician.

[29] Woldeyes DH, Kiros MD, Abegaz BA, Woya AA (2020). The size of anterior fontanelle and its determinants at birth among neonates in Northern Ethiopia: A cross-sectional study. Pediatr. Health Med. Ther..

[30] Hajizadeh Y (2020). Urinary paraben concentrations and their implications for human exposure in Iranian pregnant women. Environ. Sci. Pollut. Res..

[31] Jamal A (2020). Prenatal urinary concentrations of environmental phenols and birth outcomes in the mother-infant pairs of Tehran Environment and Neurodevelopmental Disorders (TEND) cohort study. Environ. Res..

[32] Sakhvidi MJZ (2021). The prospective epidemiological research studies in IrAN (PERSIAN) birth cohort protocol: Rationale, design and methodology. Longit. Life Course Stud..

[33] Amin MM, Ebrahim K, Poursafa P (2017). Development of a dispersive liquid–liquid microextraction (DLLME) method coupled with GC/MS as a simple and valid method for simultaneous determination of phthalate metabolites in plasma. Int. J. Environ. Anal. Chem..

[34] Fernández MF (2021). Determination of bisphenols, parabens, and benzophenones in placenta by dispersive liquid-liquid microextraction and gas chromatography-tandem mass spectrometry. Chemosphere.

[35] Vela-Soria F, Ballesteros O, Zafra-Gómez A, Ballesteros L, Navalón A (2014). A multiclass method for the analysis of endocrine disrupting chemicals in human urine samples. Sample treatment by dispersive liquid-liquid microextraction. Talanta.

[36] Vela-Soria F, Ballesteros O, Zafra-Gómez A, Ballesteros L, Navalón A (2014). UHPLC-MS/MS method for the determination of bisphenol A and its chlorinated derivatives, bisphenol S, parabens, and benzophenones in human urine samples. Anal. Bioanal. Chem..

[37] Girdhar S, Sharma S, Chaudhary A, Bansal P, Satija M (2016). An epidemiological study of overweight and obesity among women in an Urban area of North India. Indian J. Community Med..

[38] Etzel TM (2017). Urinary triclosan concentrations during pregnancy and birth outcomes. Environ. Res..

[39] Nieminen P, Lehtiniemi H, Vähäkangas K, Huusko A, Rautio A (2013). Standardised regression coefficient as an effect size index in summarising findings in epidemiological studies. Epidemiol. Biostat. Public Health..

[40] Richards JM (1982). Standardized versus unstandardized regression weights. Appl. Psychol. Meas..

[41] Hosseini SN (2014). Cosmetic products use intention among Iranian female college students. Int. Sci. Index.

[42] Zhao H (2017). Exposure to benzophenones, parabens and triclosan among pregnant women in different trimesters. Sci. Total Environ..

[43] Gao CJ (2015). Benzonphenone-type UV filters in urine of Chinese young adults: Concentration, source and exposure. Environ. Pollut..

[44] Moos RK (2014). Rapid determination of nine parabens and seven other environmental phenols in urine samples of German children and adults. Int. J. Hyg. Environ. Health.

[45] Wang L, Kannan K (2013). Characteristic profiles of benzonphenone-3 and its derivatives in urine of children and adults from the United States and China. Environ. Sci. Technol..

[46] Song S (2020). Occurrence and transfer of benzophenone-type ultraviolet filters from the pregnant women to fetuses. Sci. Total Environ..

[47] Chen H (2021). Characteristics of exposure to multiple environmental chemicals among pregnant women in Wuhan, China. Sci. Total Environ..

[48] Jiang Y (2019). Prenatal exposure to benzophenones, parabens and triclosan and neurocognitive development at 2 years. Environ. Int..

[49] Deurenberg P, Deurenberg-Yap M, Guricci S (2002). Asians are different from Caucasians and from each other in their body mass index/body fat per cent relationship. Obes. Rev..

[50] Nasab H, Rajabi S, Mirzaee M, Hashemi M (2022). Association of urinary triclosan, methyl triclosan, triclocarban, and 2,4-dichlorophenol levels with anthropometric and demographic parameters in children and adolescents in 2020 (case study: Kerman, Iran). Environ. Sci. Pollut. Res..

[51] Messerlian C (2018). Preconception and prenatal urinary concentrations of phenols and birth size of singleton infants born to mothers and fathers from the Environment and Reproductive Health (EARTH) study. Environ. Int..

[52] Ferguson KK (2018). Environmental phenol associations with ultrasound and delivery measures of fetal growth. Environ. Int..

[53] Philippat C (2013). Prenatal exposure to environmental phenols: Concentrations in amniotic fluid and variability in urinary concentrations during pregnancy. Environ. Health Perspect..

[54] Liu H (2019). Blood pressure changes during pregnancy in relation to urinary paraben, triclosan and benzophenone concentrations: A repeated measures study. Environ. Int..

[55] Huo W (2016). The relationship between prenatal exposure to BP-3 and Hirschsprung’s disease. Chemosphere.

[56] Meeker JD (2013). Distribution, variability, and predictors of urinary concentrations of phenols and parabens among pregnant women in Puerto Rico. Environ. Sci. Technol..

[57] Watanabe Y (2015). Metabolism of UV-filter benzophenone-3 by rat and human liver microsomes and its effect on endocrine-disrupting activity. Toxicol. Appl. Pharmacol..

[58] Philippat C (2014). Prenatal exposure to phenols and growth in boys. Epidemiology.

[59] Pan X, Yan L, Qu R, Wang Z (2018). Degradation of the UV-filter benzophenone-3 in aqueous solution using persulfate activated by heat, metal ions and light. Chemosphere.

[60] Chen M (2013). Association of exposure to phenols and idiopathic male infertility. J. Hazard. Mater..

